# Prevalence and determinants of stunting in under-five children in central Tanzania: remaining threats to achieving Millennium Development Goal 4

**DOI:** 10.1186/s12889-015-2507-6

**Published:** 2015-11-21

**Authors:** Innocent Antony Semali, Anna Tengia-Kessy, Elia John Mmbaga, Germana Leyna

**Affiliations:** Department of Epidemiology and Biostatistics, School of Public Health and Social Sciences, P.O. Box 65014, Dar es Salaam, Tanzania; Department of Community Health, School of Public Health and Social Sciences, P.O. Box 65014, Dar es Salaam, Tanzania

**Keywords:** Stunting, Child malnutrition, Under-five children, Millennium development goals

## Abstract

**Background:**

The Millennium Development Goal No 4 (MDG 4) requires countries to scale up interventions addressing malnutrition and other immediate determinants of burden of disease among children to reduce child mortality by two thirds by 2015, which is this year. Whereas globally some achievements have been registered, under-nourishment remains a significant problem in some developing countries such as Tanzania. This study set out to estimate the extent of stunting and its associated determinants to assess the progress made thus far towards achieving MDG 4 in Tanzania.

**Methods:**

A random sample of 678 households with under-five children was selected from two randomly selected wards of Kongwa district in Dodoma region, Tanzania. The WHO anthropometric calculator, which computes Z-scores using a reference population, was used to process the anthropometric measurement data taken from all the participants. Children with height for age Z-score of less than 2 were categorised as stunted and coded as 1 and the rest were coded as 0. Proportions of stunting were compared using the chi-square test to determine the association between stunting and the independent variables. Multivariate logistic regression analysis was carried out to determine the Adjusted Odds Ratio (AOR) of the independent determinants of stunting. The cut-off for significant association was set at *p* = 0.05. All these analyses used the STATA 12 software.

**Results:**

About half (49.7 %) of the children were stunted. This stunting was associated with belonging to households where the head of family was young (<35 years) (AOR = 0.67, 95 % CI 0.47–0.96, *p* = 0.031), young age of the mothers (AOR = 1.54, 95 % CI 1.06–2.24, *p* = 0.023), and economic variables such as owning a cellular phone (AOR = 0.66, 96 % CI 0.46–0.94, *p* = 0.023).

**Conclusions:**

Stunting was highly prevalent in Kongwa district despite general improvements in child nutritional status at the national level. Household characteristics and economic status were found to play a major role in child health. In this regard, disaggregated analyses are therefore important in identifying resilient areas in need of concerted efforts for the MDG 4 to be achieved nationwide.

## Background

Protein energy malnutrition (PEM) accounts for 1.4 % of the remaining global burden of disease estimated at 2.45 billion DALYs [1]. PEM measured anthropometrically is expressed as wasted, stunted or both. Stunting is a linear growth retardation resulting from long-term chronic under-nutrition manifesting as faltering growth [2]. Globally, it is estimated that stunting declined from 37.9 in 1990 to 26 % in 2010 and recently to 25 % [3]. Despite the aforementioned global gains, stunting in sub-Saharan Africa has plateaued at 40 % with some countries reporting even higher prevalence [4, 5]. Tanzania is ranked third among countries leading in stunting in Sub-Saharan Africa with a prevalence of 42 % [6].

Chronic malnutrition and the resultant stunting are associated with increased child morbidity and mortality, reduced physical capacity, reduced economic productivity and poor school performance [7, 8]. The ecology of stunting differs between and within countries, depending on underlying factors such as weather, policy, pricing of food, access to social services, farming practices and income level [9, 10]. Intermediate factors include household and social demographic characteristics such as household size and economic status. Immediate causes include the child’s age, gender, feeding practices, food preparation and overall health status [11, 12]. Understanding the role of these factors in causing stunting in children and hence heightened child morbidity is, therefore, important to alleviating this problem.

The global response to the alleviation of poverty and its associated high burden of disease and under-nutrition culminated in the adoption of the Millennium Development Goals (MDGs) [13]. Among health-related MDGs, MDG 4 focuses on reducing child mortality by two thirds between 1990 and 2015. One of its indicators aims to cut in half cases of underweight among under-five children. In this regard, Tanzania was projected to reduce underweight cases by half (from 28.8 % to 14.4 %) and stunting by half (from 46.6 % to 23.3 %) among under-five children by 2015 [14].

In 2013, two years from the final timeline (2015) a United Nations report revealed that the proportion of undernourished children in developing regions had declined by 35.8 % from 1990 to 2012 [6]. On the other hand, developing countries such as Tanzania had only recorded a reduction of two percent in the same period [6]. As the target deadline approaches, determining reasons for this slow progress is essential in strategising and developing remedial measures. Such data will inform post-MDG 4 efforts towards addressing child malnutrition using stunting as a proxy indicator. The aim of this study, therefore, was to provide information on factors that influence stunting which are crucial to understand in the process of planning for achievement in the post-MDG era.

## Methods

The study was approved by the Muhimbili University of Health and Allied Sciences (MUHAS) Institutional Review Board (IRB). A cross-sectional study was then conducted in Kongwa district, one of the six districts in Dodoma region (province), located in central Tanzania. The district is divided into 22 administrative wards, each of which is further sub-divided into 3-5 villages. The population of the district is estimated at 300,000 people. The district is served by one public hospital, three health centres and eight dispensaries.

Sample size calculation considered the national stunting prevalence rate of estimated at 42 % [15], power 90 % and 0.05 precision yielding 688 as the a sample size. We randomly selected two wards out of the 22 wards in the district. The first one, Mlali, had a population of 19,623 and the second, Ugogoni, had 17,048 people. A sampling frame consisted of households with an under-five child. Once compiled, the sampling frame was subjected to random selection of the required number of households from each village (with the sample size corrected for the sampling design). Equal numbers of households were selected from each ward. Within a household, both the mother and the head of the household were interviewed and anthropometric measurements for under-five children were taken.

### Data collection tools

Field data were collected using a pretested structured interview guide that was administered by trained research assistants. The interview guide collected information on the socio-demographic characteristics, ownership of selected assets, distance to the health facility and time to the nearest water source.

Permission was obtained from the mother prior to weighing the child using Salter scales. All the children were weighed wearing minimal clothing and without shoes. The weight was recorded to the nearest 100 grams and the height to the nearest 1 mm [16]. Each day and before any weighing, the scale was adjusted to zero for accuracy and consistency by using a 2 kg weight. A pair of trained research assistants assisted by the mother or guardian used a length measurement board with a sliding headpiece to measure the height of each child. For children under the age of two years as well as children who were unable to stand upright, their recumbent height was determined using a length board placed on a flat and firm surface. Such children were then placed lying supine along the middle of the board. Appropriate records were entered accordingly into the child’s checklist, which was checked for completeness and consistency at the end of the day.

### Data management and analysis

The data were checked for completeness and consistency before being entered into the computer using EPI-INFO 3.2.3 database. Data cleaning was done and then followed by the generation of frequencies and descriptive statistics using STATA version 12 [17]. Independent variables included individual and community level variables. *Individual* variables included the age and education of the mother and the head of household, the number of children in the household and the marital status of the mother. *Community (place)* variables included distance to the nearest health facility and time to the nearest water source. The WHO Emergency Assessment Tool was used to calculate Z-scores using the anthropometric data of each child. Those with less than minus two (-2) Z-scores were classified as stunted and were coded as 1 and the rest were coded as 0.

From the outset, comparison of percentages of stunted children by categories of the independent variables was done using the chi-square test to determine the significance of the difference observed. A multivariate logistic model that entered all 12 independent variables in one single step was adopted. The multivariate logistic regression determined the Adjusted Odds Ratio (AOR) for the association of the dependent and independent variables controlling for the rest of the variables. Significance level was set at *p*-value 0.05 or less.

Prior to their participation, the aim and benefits of the study were explained clearly to the parents in addition to their right to voluntary participation. Respondents were also given an opportunity to ask questions for clarification purposes and when it was clear they had understood the nature of the study and their rights, each participant was requested to provide a written consent; only those who consented were recruited for participation in the study.

## Results

The analysis involved 678 under-five children. More than half (55.6 %) of these children were from households headed by persons under 35 years; and close to a third of the households (32.2 %) were female-headed (Table 1). About a fifth (21.5 %) of the mothers were not married, and close to a third (31.1 %) of the heads of the household had not completed primary school education. The data further show that more than half (57.4 %) of the households surveyed had five or more persons. A large proportion (81.6 %) of the respondents were from households that had to walk five or more kilometres to the nearest health facility and three-quarters (75.1 %) of them walked for 15 minutes or more to a water source.

**Table 1:** Social demographic and economic characteristics of the study population

Characteristic	Variable	N	%
Age of the head of household (years)			
	<35	377	55.6
	35+	301	44.4
Sex of the head of Household			
	Female	218	32.2
	Male	460	67.8
Respondent marital status			
	Married	526	78.5
	Un-married	144	21.5
Education of the head of Household (years)			
	7 years or more	467	68.9
	Less than 7 years	211	31.1
Mothers education			
	7 years of more	389	57.4
	Less than 7 years and more	289	42.6
Mothers age group			
	<25	217	32.0
	25+	461	68.0
Number of children			
	<5	542	79.9
	5+	136	20.1
Number of people in household			
	<5	289	43.6
	5+	389	57.4
Distance to health facility			
	<5	125	18.4
	5+	553	81.6
Time to water sources (minutes)			
	<30	169	24.9
	30+	509	75.1
Size of farm owned (acres)			
	<5	549	81.0
	5+	129	19.0
Own livestock			
	Yes	339	50.0
	No	339	50.0

About half (49.7 %) of the children were stunted with variations dependent on their household characteristics. Stunting was higher (56.1 %) among children from households whose mothers had little formal education compared to those (44.9 %) whose mothers had completed class seven or higher (see Table 2). This difference was statistically significant (χ^2^ = 8.126, *p* = 0.004). Additionally, stunting was significantly less among children residing in households located less than five kilometres from a health facility (39.2 %) compared to their counterparts (52.1 %) residing further away (χ^2^ = 6.675, *p* = 0.009).

**Table 2:** Bivariate analysis of social demographic characteristics and stunting

Characteristic	Attribute	N	Stunted (%)	Chi-square	*p*-value
Age of the head of the Household (years)					
	<35	377	46.7		
	35+	301	53.5	3.099	0.078
Sex of the head of the household (years)					
	Female	218	46.8		
	Male	460	51.1	1.092	0.296
Respondent married					
	Married	526	50		
	Un-married	144	49.3	0.0218	0.883
Education of the head of the Household					
	7 years or more	467	48.6	0.722	
	Less than 7 years	211	52.1		0.395
Mothers education					
	7 years of more	389	44.9		
	Less than 7 years and more	289	56.1	8.126	0.004
Mothers age group					
	<25	217	54.4		
	25+	461	47.5	2.788	0.095
Number of children					
	<5	542	48.5		
	5+	136	54.4	1.508	0.220
Number of people in household					
	<5	289	48.8		
	5+	389	50.4	0.169	0.681
Distance to health facility					
	<5	125	39.2		
	5+	553	52.1	6.675	0.009
Time to water sources					
	<30	169	43.2		
	30+	509	51.9	3.816	0.051
Size of farm owned					
	<5	549	51.0		
	5+	129	44.2	1.941	0.164
Livestock					
	Yes	339	48.7		
	No	339	50.7	0.289	0.591

Multivariate logistic analysis as presented in Table 3 shows that independent factors were significantly associated with reduced odds of stunting. These factors included young age (less than 35 but older than 25) of the head of household (OR = 0.67, 95 % CI 0.47–0.96, *p*-value = 0.031), high mother’s education (OR = 0.71, 95 % CI 0.51-1, *p*-value = 0.05), and ownership of a mobile phone (OR = 0.66, 95 % CI 0.45–0.94, *p*-value = 0.023). However, a young age of less than 25 years of the mother increased the likelihood of stunting (OR = 1.54, 95 % CI 1.06–2.24, *p*-value = 0.023).

**Table 3:** Multivariate logistic regression analysis of stunting against selected characteristics

Characteristic		Odds Ratio	95 % Conf. Int.	*p*-value
Age of the head of the Household (years)	<35	1		
	35+	0.67	0.47–0.96	0.031
Sex of the head of the household	Male	1		
	Female	1.28	0.86–1.92	0.224
Mothers age	25+	1		
	<25	1.54	1.06–2.24	0.023
Mother is Married	Yes	1		
	No	0.93	0.59–1.46	0.743
Mothers education (years)	<7	1		
	7+	0.71	0.51–1.00	0.050
Education of the head of the Household (years)	<7	1		
	7 +	1.08	0.75–1.55	0.692
Number of children	<5	1		
	5+	0.81	0.52–1.24	0.334
Number of people in households	<5	1		
	5+	1.01	0.71–1.43	0.964
Owned a bike	No	1		
	Yes	0.76	0.55–1.07	0.117
Owned a hand phone	No	1		
	Yes	0.66	0.46–0.94	0.023
Owned livestock	Yes	1		
	No	1.01	0.71–1.43	0.964
Owned farms	Yes	1		
	No	1.17	0.77–1.78	0.461
Distance to health facility	5 +	1		
	<5	0.77	0.49–1.18	0.232
Time to water source	30+	1		
	<30	0.69	0.48–1.01	0.058

## Discussion

About half (49.7 %) of the under-five children who took part in the study conducted in Kongwa district, central Tanzania, were stunted, thus presenting a significant public health problem. Factors significantly associated with reduced odds of stunting included if mothers education was more than seven years, age of the head of household being less than 35 years and ownership of a mobile phone. The prevalence of stunting observed was slightly higher than the estimated national average of 42 % [6]. This finding supports the observation that Tanzania is one of the leading countries in terms of stunting, ranked third after Ethiopia and the Republic of Congo [9]. Although some of the regions with very high proportions of stunting are paradoxically the same areas leading in food production in Tanzania, Kongwa district’s case was different. Kongwa district is one of the areas in central Tanzania that often receives low rainfall and consequently, poor harvests.

In this study, children of young mothers (aged less than 25) were more likely to be stunted than those of older mothers. This is contrary to the findings in Ghana where stunting was associated with advanced maternal age (30–44 years) [2]. A possible explanation for such an association could be that younger mothers are more likely to have several young children and, therefore, have to stretch their food supply even farther. In addition, young mothers might have limited access to socio-economic resources to meet the nutritional needs of their children.

The study also found that children whose mothers had modestly higher education levels were significantly less likely to be stunted than those of mothers with less or no formal education. In fact, an educated mother is more likely to have a better income base through better employment opportunity and efficient management of income generation activities than an uneducated or less educated one and thus is better able to meet the nutritional needs of her family. Likewise, an educated mother would have better skills and better information for planning purposes as well as for implementing strategies that can meet adequately the nutritional needs of her children and the whole family [9]. Related studies elsewhere similarly reported a strong relationship between maternal education levels and reduced likelihood of stunting [10, 11, 18]. Also mothers with better education will start giving birth at a relatively older age as they stay in school longer than less educated women. Consequently, they are likely to have acquired better childcare skills over time that is essential to meeting the nutritional needs of their children.

Moreover, children from households where a member owned a cell phone were less likely to be stunted. Owning or operating a mobile phone is a proxy measure of economic status, implying a better ability to meet the needs of the family. Studies elsewhere also support the relationship between economic wellbeing and stunting [10, 11, 19]. In this regard, owning a mobile phone facilitates communication, and sustains social networks that could be contacted to provide support in case of food shortages. Such electronic gadgets also facilitate accessing information that can enhance the household’s capacity to mitigate food insecurity, thus prevent stunting.

As this was a cross-sectional study, it was limited in its ability to determine a causal relationship between the observed factors and stunting. However, most of the factors observed have already been proven by other study designs with strong plausibility and consistency. On the other hand, reporting bias, which could have affected the findings in this study has been reduced by the use of well-trained primary school teachers in data collection. Since stunting is a physical measurement, it was not vulnerable to subjectivity, implying that the prevalence of stunting observed has good internal validity.

## Conclusions

Therefore, we conclude that despite the national level improvement in child nutrition status, stunting was highly prevalent in Kongwa district and that household characteristics as well as the economic status of individual households do play a major role in child health promotion and stunting prevention. Disaggregated analyses are, therefore, important in identifying vulnerable areas in need of concerted efforts to engender the achievement of MDG 4 nationwide.

## Abbreviations

AOR: Adjusted Odds Ratio
DALLYs: Disability Adjusted Life Years Lost
MDG4: Millennium Development Goal number 4
MUHAS: Muhimbili University of Health and Allied Sciences
PEM: Protein Energy Malnutrition
SIDA/SAREC: Swedish International Development Co-operation Agency/Swedish Agency for Research Co-operation
WHO: World Health Organisation
